# Potentially toxic elements pollution in road deposited sediments around the active smelting industry of Korea

**DOI:** 10.1038/s41598-021-86698-x

**Published:** 2021-03-31

**Authors:** Hyeryeong Jeong, Jin Young Choi, Kongtae Ra

**Affiliations:** 1 Marine Environmental Research Center, Korea Institute of Ocean Science and Technology (KIOST), Busan, 49111 Korea; 2 Department of Ocean Science (Oceanography), KIOST School, University of Science and Technology (UST), Daejeon, 34113 Korea

**Keywords:** Environmental sciences, Environmental chemistry, Environmental impact

## Abstract

Potentially toxic elements (PTEs) were investigated in the different sizes of road deposited sediments (RDS) around the active smelting industry to understand their sources and to assess the pollution and ecological risk levels. The highest PTEs concentrations was shown near the raw materials import port and the smelting facilities. The fine particles of RDS showed extremely high PTEs concentrations. Zn has the highest mean concentration in the < 63 μm particle size of RDS, followed by Pb > Cu > As > Cr > Ni > Cd > Hg. The PTEs concentrations of this study were the highest values compared to the soils around the smelter and the RDS in urban and industrial areas in the world. This indicates that these PTEs pollution in RDS were mainly attributed to the transportation of raw materials for the smelting industry. According to nemerow pollution index calculation, RDS at all sampling sites with particles of less than 250 mm was seriously polluted with PTEs. The ecological risk was also found to be very high in all RDS fractions and highly toxic elements such as Cd, Pb and Hg pose extremely risk. Given the total amounts PTEs in the road surface, it is necessary to apply RDS removal management plan to reduce the PTEs pollution.

## Introduction

Road infrastructure and transportation are important components of urban areas and have enabled the rapid development of industrialization and urbanization. Road deposited sediments (RDS) are highly contaminated with potentially toxic elements (PTEs) by various traffic and industry-related sources such as vehicular exhaust and non-exhaust sources, atmospheric deposition and surrounding soil erosion and spill of industrial raw materials during transportation^1–6^. Thus, roads are often a prominent point-source and non-point source of dissolved and sediment-associated PTEs^6–10^. The particle size distribution of RDS is a very important factor as it determines the behavior and mobility of the particles and shows the highest concentration of PTEs in fine particles^11–13^. Environmental concern related to RDS is that RDS containing high concentrations of PTEs on the road surface adversely affects the surrounding environments as well as human health^14–16^. The fine fractions of RDS are readily transported to the surrounding aquatic environments by stormwater runoff. Many studies reported that the fine particle (< 44 μm^17^, < 63 μm^18^, < 125 μm^6^) largely contributed of total suspended solids (TSS) load in stormwater runoff from urban and industrial areas. The finer RDS are also re-suspended by strong winds and the high-speed movement of the vehicles, therefore, PTEs bound to fine particles of RDS and surrounding soils can enter the human body via inhalation, ingestion and dermal absorption^19–21^. Our previous study reported that 14.3–15.8 g/m^2^ (< 63 μm) and 3.2–4.2 g/m^2^ (> 1000 μm) of RDS in urban area accumulate on the road surface in Korea^22^. On road surface, RDS are deposited of 11.7 g/m^2^ in urban area^17^ and 174.6 g/m^2^ in industrial area^6^. Industrial areas are characterized by a higher accumulation of road dust than urban areas. In Korea, the amounts and concentrations of PTEs in fine particle of RDS were much higher in industrial area than in urban areas^5,22,23^. Given the total length of road, a huge amount of PTEs would have been accumulated in road surface. Of course, PTEs pollution in industrial RDS is subjected to the complex influence of traffic and industrial activities. Lanzerstorfer^13^ reported that the PTEs concentrations in urban RDS can be used as a useful indicator for environmental pollution. The potential sources of PTEs can be identified by evaluating the PTEs concentrations in RDS from different land use types and the elemental ratios of them^11,24–26^. Although there are very few RDS studies in industrial areas compared to urban areas, the study of PTEs concentration in RDS from the industrial area will make it possible to differentiate between transport and industrial activities. The objectives of this study are to: (1) evaluate the PTEs pollution levels of different RDS sizes in the industrial area where the smelting industry is active; (2) identify the pollution sources of PTEs; (3) assess the potential ecological posed by PTEs.

## Materials and methods

### Sampling and PTEs analysis

**Figure 1:**
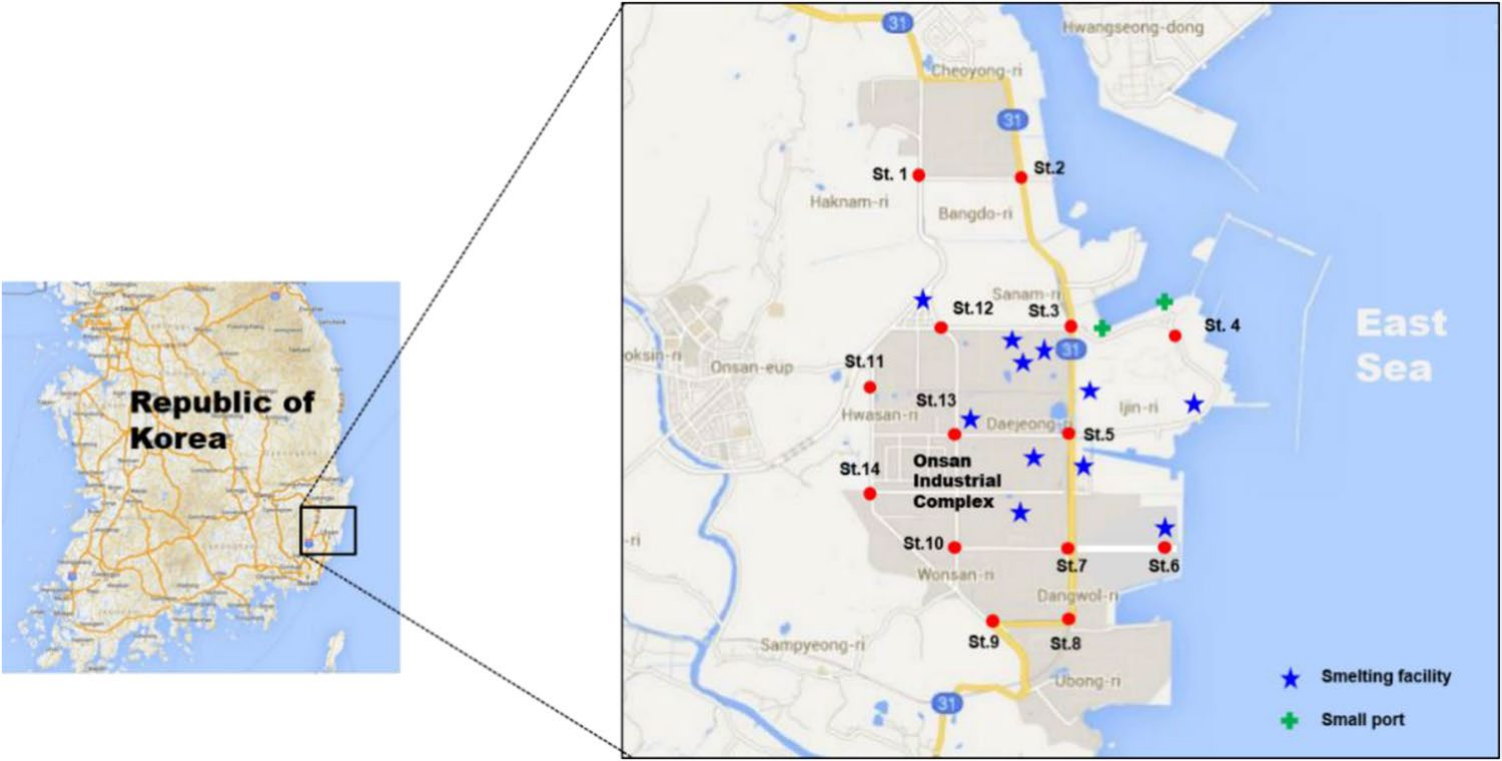
Locations of sampling sites for road deposited sediments from Onsan industrial complex including the smelter of Korea. Figure created using Microsoft Office PowerPoint 2016 based on Google Maps (available at https://www.google.com/maps).

Total of 14 RDS samples were collected from Onsan Industrial complex including several smelting facilities of Korea (Fig. 1) during December 2013 following a dry weather periods of about 10 days. Average temperature, humidity, and wind speed were 4.3 °C, 52.0%rh, and 6.7 m/s respectively. The RDS were collected in four and more sub-sampling for each site using a cordless vacuum cleaner (DC-35, Dyson Co., UK) with 0.5 m × 0.5 m space along the curb of the road. This vacuum cleaner can collect dust with high efficiency using powerful centrifugal forces spin. After collecting RDS samples, the vacuum cleaner was disassembled and cleaned, and the parts that were difficult to clean were replaced with new ones to prevent cross-contamination. Each RDS sample were sieved individually using < 63 μm, 63–125 μm, 125–250 μm, 250–500 μm, 500–1000 μm, > 1000 μm^27^ by using vibratory sieve shaker (Analysette 3 pro, Fritsch Co., Germany) with nylon sieves in laboratory. Each fraction of RDS sample was weighted, pulverized (Pulverisette 6, Fritsch Co., Germany) and stored separately into pre-acid cleaned polyethylene bottle until metal analysis. The weight (g) of each RDS size fraction accounted for 7.3% (< 63 μm), 11.8% (63–125 μm), 23.2% (125–250 μm), 31.8% (250–500 μm), 17.1% (500–1000 μm), and 8.9% (> 1000 μm) of the total RDS samples. About 0.1 g of each ground and homogenized RDS sample was weighted in Teflon digestion vessel added with high purity (Ultra-100 grade, Kanto Chemical, Japan) of HNO_3_, HF and HClO_4_ on a hot plate at 180 °C for 24 h for total digestion. After evaporation and redissolution with 2% HNO_3_, heavy metals of Cr, Ni, Cu, Zn, As, Cd and Pb were analyzed using inductively coupled plasma mass spectrometry (ICP-MS, iCAP-Q, Thermo Scientific Co., Germany). Hg was determined using Hg analyzer (Hydra-C, Leeman Labs, USA) based on the USEPA 7473 method. The blanks and duplicate measurements were performed for quality control. Two types of certified reference materials for MESS-4 and PACS-3 (National Research Council, Canada) were used to check data accuracy. Recoveries ranged between 96.4% and 102.1% for MESS-4 and between 93.9% and 106.0% for PACS-3, respectively.

### Pollution level assessment

The geo-accumulation index (I_geo_), proposed by Muller^28^, can be used to assess the pollution level of individual metal using the following equation:

$${\text{I}}_{{{\text{geo}}}} = \log_{2} ({\text{C}}_{{\text{i}}} /(1.5 \times {\text{B}}_{{\text{i}}} ))$$

where C_i_ and B_i_ are the concentrations of RDS samples and the geochemical background values^29^, 1.5 is the background correction efficient. I_geo_ value were classified into seven categories^28,30^.

The nemerow index (P_N_) are widely used to make a comprehensive evaluation of the pollution levels of heavy metals in soils and sediments^31–35^ and was calculated using the following equation:

$$P_{N} = \sqrt {\frac{{\left( {\frac{1}{n}\sum\nolimits_{i = 1}^{n} {PI_{i}^{2} } } \right) + \left( {Max\,PI} \right)^{2} }}{2}}$$

where PI represent a single pollution index of metal *i*, PI_i_ = C_i_/S_i_. C_i_ is the measured concentration of each metal *i*. The calculated results of P_N_ using the geological background value can be overestimated the magnitude of metal pollution^36^. Therefore, the soil quality guideline values were used in this study to better reflect the comprehensive pollution level of heavy metals in Korea. S_i_ is the soil pollution concern standard for road and factory site in Korea and its values (mg/kg) of Cr^6+^, Ni, Cu, Zn, As, Cd, Pb and Hg were 40, 500, 2000, 2000, 200, 60, 700 and 20, respectively^37^. In Korea, soil samples are sieved with a 150 μm mesh. PTEs in soils (< 150 μm) are analyzed and compared with the soil pollution concern standard. In case of Cr, in Korea soil quality guideline, the concentration of Cr^6+^ is recommended. Lazo^38^ reported that the content of Cr^6+^ accounts for more than 90% of total Cr in the contaminated area. Therefore, the application of total Cr concentration instead of Cr^6+^ of this study did not significantly affect the results of pollution evaluation for eight metals using P_N_. This index divides pollution into five grades^39^.

### Potential ecological risk assessment

Potential ecological risk index (PER), proposed by Hakanson^40^ can be used to assess the risk of eight metals based on their toxicity response using the following equations:

$$\begin{aligned} E_{r}^{i} & = T_{r}^{i} \times \left( {C_{i} /B_{i} } \right) \\ PER & = \sum\limits_{i = 1}^{n} {E_{r}^{i} } \\ \end{aligned}$$

where C_i_ and B_i_ were the same as those in I_geo_ calculation. ${\text{E}}_{{\text{r}}}^{{\text{i}}}$ is the single factor ecological risk degree for PTEs. ${\text{T}}_{{\text{r}}}^{{\text{i}}}$ is the toxic response factor for a single metal pollution (Hg = 40, Cd = 30, As = 10, Cu = Ni = Pb = 5, Cr = 2, Zn = 1)^40,41^. ${\text{E}}_{{\text{r}}}^{{\text{i}}}$ were classified into five classes^42^ and the PER value were classified into four classes^40,43^. PASW statistics program (version 18) was used for the Pearson’s correlation analysis and principal component analysis (PCA) to extract correlation among PTEs in this study. Hierarchical cluster analysis (HCA) was also performed to understand the relationship between different size fractions of RDS.

### Grain size fraction loading

The grain size fraction loading (GSF_loading_) was calculated using the relative mass loads of PTEs in the six particle size fractions of the RDS, which is expressed as follows:

$$GSF_{loading} = 100 \times \left[ {\frac{{X_{i} \times GS_{i} }}{{\mathop \sum \nolimits_{i = 1}^{6} X_{i} \times GS_{i} }}} \right]$$

where X_i_ is the concentration of PTEs by particle size fraction separated from each RDS sample, and GS_i_ is the mass percentage of each particle size fraction. The sum of the GSF_loading_ values for each RDS sample is always 100%^44^.

## Results and discussion

### PTEs contents in different sizes of RDS

**Figure 2:**
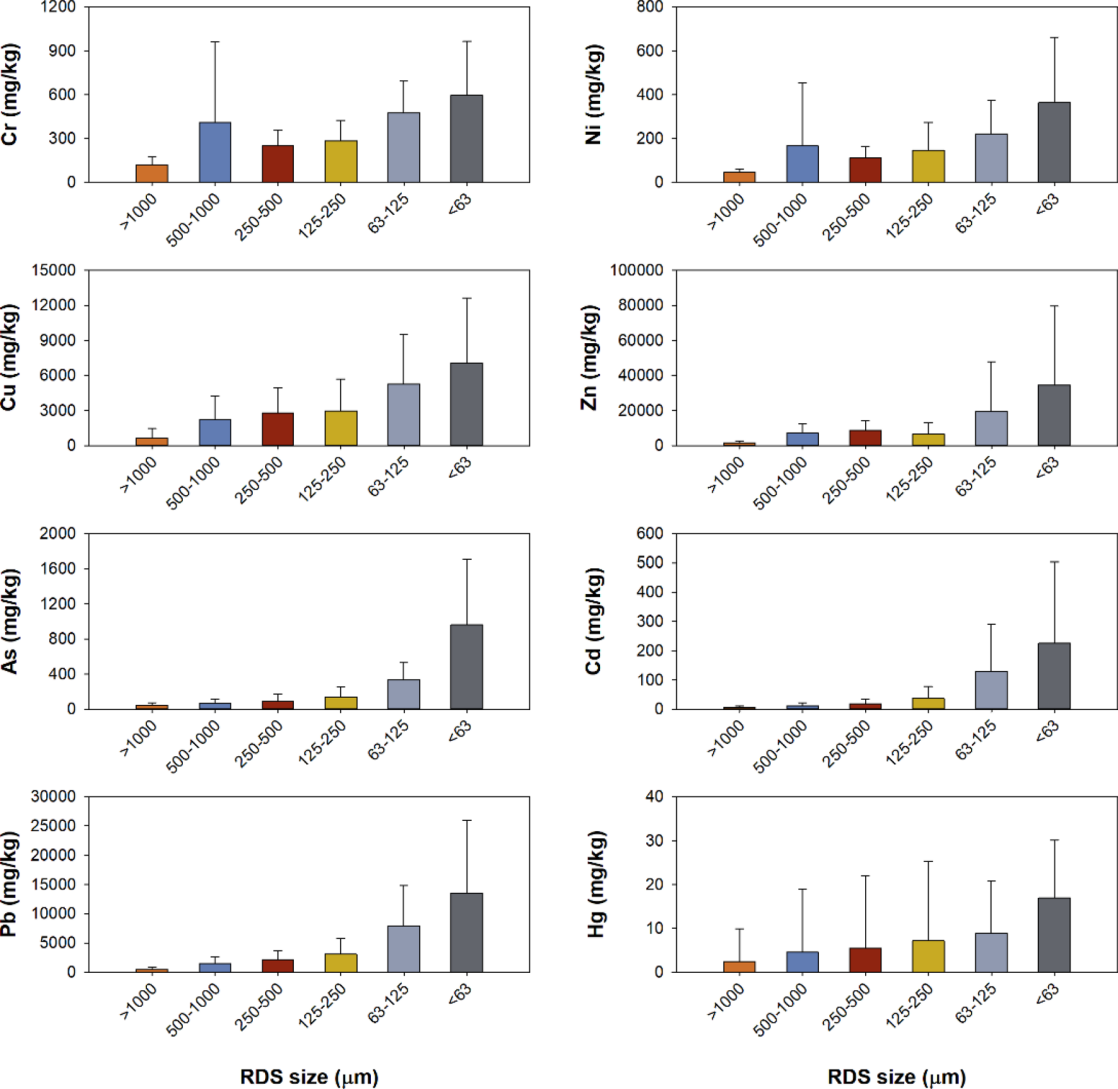
Comparison of mean PTEs concentrations (mg/kg) with standard deviation in the different sizes of road deposited sediments of this study.

The minimum, maximum and mean values of the total RDS amount and Cr, Ni, Cu, Zn, As, Cd, Pb, and Hg concentrations are shown in Table 1. Crustal elements such as Al, Fe, and Li showed no significant difference depending on different sizes of RDS (Table S1). The Cu, Zn, As, Cd, Pb, and Hg concentrations significantly increased with decreasing in particle size of RDS (Fig. 2). Mean PTE concentrations in the fine particle size (< 63 μm) of RDS was 5.0 (Cr) ~ 55.5 (Zn) times higher than those in the large particle size (> 1000 μm). The mean concentration of RDS (63 μm) was highest for Zn at 34,592 mg/kg, followed by Pb (13,561) > Cu (7071) > As (961) > Cr (596) > Ni (364) > Cd (225) > Hg (17). The Cr and Ni concentrations in the fine particle size (< 63 μm) showed highest values at S6 site, but the highest concentrations for Cu, Zn, As, Cd were observed in S4 and S5 sites which the smelting facilities exist (Fig. S1 and S2).

**Table 1:** Minimum, maximum, and mean values of PTEs in the different sizes of road deposited sediments of this study.

Size (μm)	RDS (g/m^2^)	Cr (mg/kg)	Ni (mg/kg)	Cu (mg/kg)	Zn (mg/kg)	As (mg/kg)	Cd (mg/kg)	Pb (mg/kg)	Hg (mg/kg)
** > 1000**									
Min	4	51	26	71	256	3	0.9	65	0.01
Max	298	220	68	3212	3735	100	20.9	1685	27.9
Mean	104	120	46	623	623	46	6.3	470	2.5
SD	92	56	15	846	846	28	5.0	418	7.4
**500–1000**									
Min	30	76	28	249	1177	10	2.0	209	0.1
Max	688	2262	1156	7537	16,579	187	45.9	4083	54.6
Mean	200	410	166	2243	7167	67	11.5	1483	4.5
SD	193	552	287	2016	5186	49	11.2	1107	14.4
**250–500**									
Min	85	80	23	294	1518	25	2.2	240	0.1
Max	1104	435	187	8477	20,034	341	64.2	6264	62.4
Mean	373	249	111	2782	8646	92	18.8	2171	5.5
SD	330	107	50	2170	5708	85	16.9	1560	16.4
**125–250**									
Min	72	100	23	284	1279	12	3.7	268	0.3
Max	704	678	556	10,875	26,279	374	157.9	10,311	69.6
Mean	272	285	147	2978	6743	141	38.0	3072	7.2
SD	223	138	127	2708	6135	110	39.4	2718	18.1
**63–125**									
Min	53	199	54	348	2152	59	7.4	463	0.4
Max	335	921	645	16,511	112,751	840	644.1	23,536	46.4
Mean	138	476	222	5265	19,620	330	128.7	7924	8.9
SD	94	217	153	4258	28,085	204	162.6	6927	11.9
** < 63**									
Min	28	200	106	975	4400	110	17.6	1253	1.2
Max	197	1416	1081	18,873	166,457	2721	975.6	44,667	45.1
Mean	85	596	364	7071	34,592	961	225.1	13,561	17.0
SD	50	368	299	5552	44,856	744	278.6	12,438	13.2

The study area, Onsan industrial complex, has concentrated non-ferrous metal production industry of Korea. There are many smelting facilities in operation that produces 1.2 Mt of nonferrous metals annually, including Cu, Zn, Cd and Pb. The largest smelter in this study region produces high-purity ingots for Cu 25,800 t, Zn 650,100 t, and Pb 413,000 t. Garmash (1985)^45^ found that nonferrous metal smelters are more contaminated with Zn, Pb, and Cd in soils than iron smelters. The amount of RDS accumulated on road surface in the study area is higher than that in urban areas. There are raw material import ports and outdoor raw material storages for smelting industry on the north of S4 site.

The PCA results indicated that the two principal components explaining 72.498% of the total variance (Table S2). Kaiser–Meyer–Olkin (KMO) value was found to be 0.745 and Bartlett’s test value was 0 (*p* < 0.001), confirming to be suitable for PCA analysis. PC1 was dominated by Cu, Zn, As, Cd, and Pb, accounting for 50.034% of the total variance (Table S2). RDS of this study is significantly correlated with among Cu, Zn, As, Cd, and Pb. Raw materials are transported using a large truck. The highest PTEs concentrations were observed in all particle sizes of RDS around the smelting facilities, indicating that raw materials for the smelting industry were spilled onto the road surface during transportation. PC2 consisted of Cr and Ni, explaining 22.464% of the total variance (Table S2). A high correlation between Cr and Ni was observed. Cr and Ni are discharged from furnaces during the manufacture of iron and steel, or also used in alloy manufactures such as stainless steel and chromium plating^46^. Jo et al.^47^ reported that Cr and Ni contamination in roadside soil was affected by traffic and industrial activities in Korea. Generally, Cr is used in vehicle parts including metal plating, wrist pins, and connecting rods^48^. Adamiec et al.^3^ reported that the urban and motorway road dust were contaminated with Cr from the abrasion of brake and alloys (wrist pins and connecting rods). In this study, the contamination of Cr and Ni was lower than that of other metals, indicating that Cr and Ni contamination was not directly related to the smelting industry. The highest concentrations of Cr and Ni were observed at S11 site, with high traffic activity connected to the highway. Therefore, Cr and Ni were more related to traffic activities in this area.

Hierarchical cluster analysis was also conducted to understand the relationship among the different sizes of RDS. The dendrogram of the different particle sizes of RDS shows two cluster groups (Fig. S3). Group 1 comprises two particle size fractions (< 125 μm) with significant PTEs contamination. Group 2 corresponded to the particle size of < 125 μm with moderate PTEs contamination.

The PTEs concentrations of this study are higher than those of RDS in urban area of Korea ^10,22,23^, indicating that RDS of industrial area are mainly influenced by industrial activities related to transportation of raw materials for smelting industry. In particular, the concentration of PTEs in the fine (< 63 μm) size of RDS in this study were the highest values compared to the RDS in urban cities^3,22,49–51^ and the soils around the smelter^52–62^ in the world (Table 2).

**Table 2:** Comparison between the average (median value in parenthesis) PTEs concentrations (mg/kg) in the road deposited sediment (< 63 μm) and those in the other published data.

Cr	Ni	Cu	Zn	As	Cd	Pb	Hg	Sample types	References
596 (470)	364 (264)	7071 (5074)	34,592 (18,362)	961 (623)	225.1 (122.8)	13,561 (9791)	17.0 (13.2)	< 63 μm, RDS	This study (N = 14)
167 (156)	50 (49)	160 (139)	907 (955)	15.7 (14.0)	1.4 (1.1)	207 (181)	0.04 (0.05)	< 63 μm, RDS	Urban, Korea (N = 5)^22^
841 (637)	246 (268)	193 (166)	2982 (2739)	16.0 (15.7)	2.1 (1.6)	221 (159)	0.21 (0.16)	< 63 μm, RDS	Industrial, Korea^49^
	52.1	345	1271		2.3	223		< 75 μm RDS	Urban, Korea^50^
		124	630		38	350		< 63 μm, RDS	Urban, Spain^51^
182	109	287	1829		0.9	456		< 20 μm, RDS	Motorway, Poland (N = 3)^3^
		78	1062		5.54	363		Top soil (0–30 cm)	Pb/Zn smelter, Australia^52^
			7366 (8285)		102 (120)	2401 (2340)		Top soil (0–36 cm)	Pb/Zn smelter, France (N = 3)^53^
		4011	1503	333		1503		Soil (10–30 cm)	Cu smelter, Poland^54^
	(10.2)	(13.6)	(2175)	(81)	(14.8)	(545)		Top soil (0–10 cm)	Pb/Zn smelter, Poland (N = 137)^55^
		161	3630		54.5	1740		Top soil (0–15 cm)	Pb/Zn smelter, UK (N = 5)^56^
227	247	666	5917		138	4892		Top soil (0–20 cm)	Pb/Zn smelter, Bulgaria^57^
		118	2558	76	31.7	953	2.27	Top soil (0–20 cm)	Pb/Zn smelter, China (N = 9)^58^
		39	597		22.1	992		Top soil (0–5 cm)	Pb/Zn smelter, China (N = 12)^59^
		(100)	(1100)	(100)	(7.6)	(2600)	(0.85)	Surface soil	Pb/Zn smelter, Kosovo (N = 30)^60^
160 (160)	54 (54)	44 (41)	280 (210)	9.8 (9.2)	7.7 (6.4)	220 (210)	0.25 (0.28)	Top soil (0–5 cm)	Pb/Zn smelter, Macedonia (N = 159)^61^
		4575 (4550)	3380 (3550)	1780 (1650)	128 (122)	5925 (5990)		Top soil (0––10 cm)	Cu/Pb smelter, Namibia (N = 4)^62^

### Pollution assessment in industrial RDS

Based on PTEs concentrations in different particle sizes of RDS, quantification of PTEs pollution was conducted using the I_geo_ and P_N_ indices. Comparison of mean I_geo_ values in different particles size of RDS is shown in Table 3. RDS of less than 63 μm had the highest I_geo_ value for all PTEs. The mean of I_geo_ values of PTEs for < 63 μm size of RDS are arranged in the following order: Cd > Pb > Zn > Hg > Cu > As > Ni > Cr. The mean values of I_geo_ for Cr and Ni show that the large particle (> 125 μm) is not polluted, but the fine particle (< 125 μm) is characterized as medium to heavily pollution. The mean values of I_geo_ showed that the RDS less than 1000 mm have an extremely heavy pollution for Cu, Zn, Cd, and Pb. For the case of As and Hg, the mean values of I_geo_ in RDS less than 125 μm exceeded 5 corresponding extremely heavy pollution, and RDS larger than 500 μm had relatively low pollution levels.

**Table 3:**
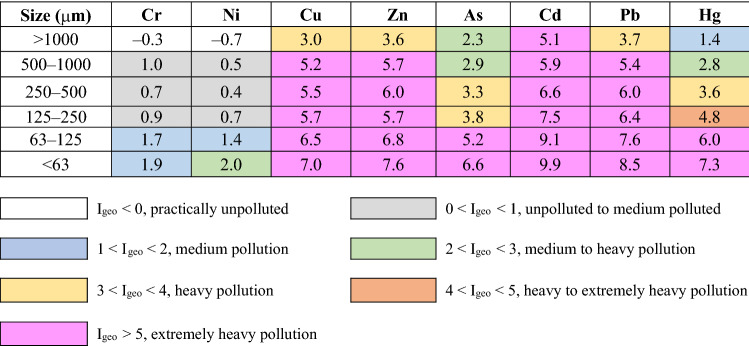
Mean values of geo-accumulation index (I_geo_) of PTEs in the different size of road deposited sediments.

**Figure 3:**
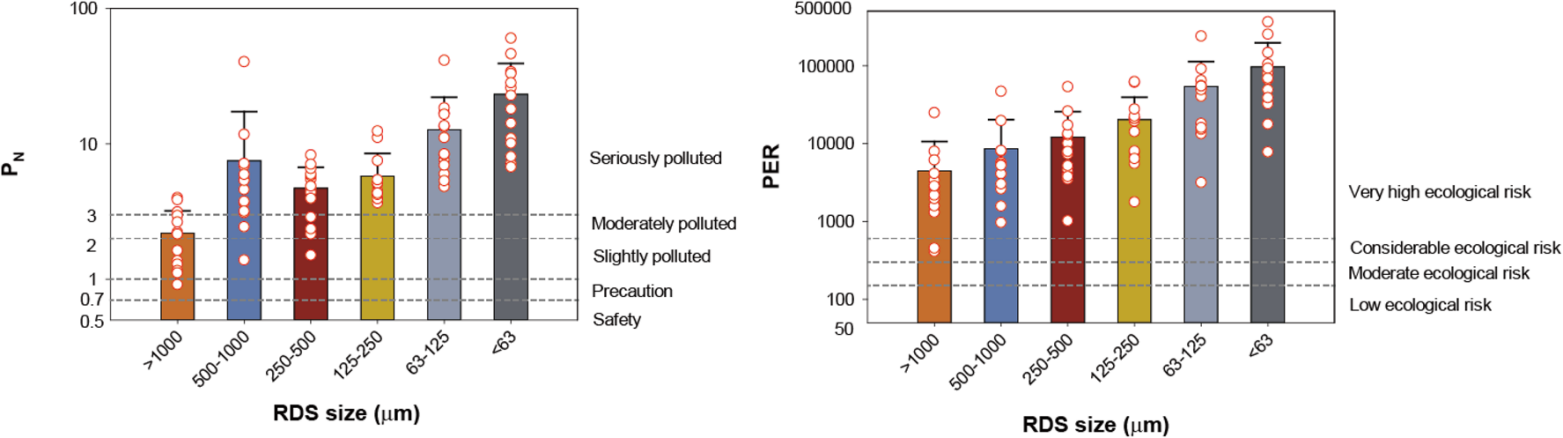
Comparison of nemerow index (P_N_) and potential ecological risk index (PER) of the difference sizes of road deposited sediments of this study.

The results of nemerow index (P_N_) showed that the mean values were in the descending order of less than 63 μm (23.2) > 63–125 μm (12.8) > 125–250 μm (5.8) > 250–500 μm (4.7) > 500–1000 μm (7.5) > above 1000 μm (2.2). As the RDS size decreased, the P_N_ value increases. Generally, fine particle sizes of RDS have high concentrations of PTEs than coarse particles^6,17^. For the RDS size less than 250 μm, P_N_ values are significantly exceeding 3 at all sampling sites, representing serious polluted with PTEs (Fig. 3).

**Figure 4:**
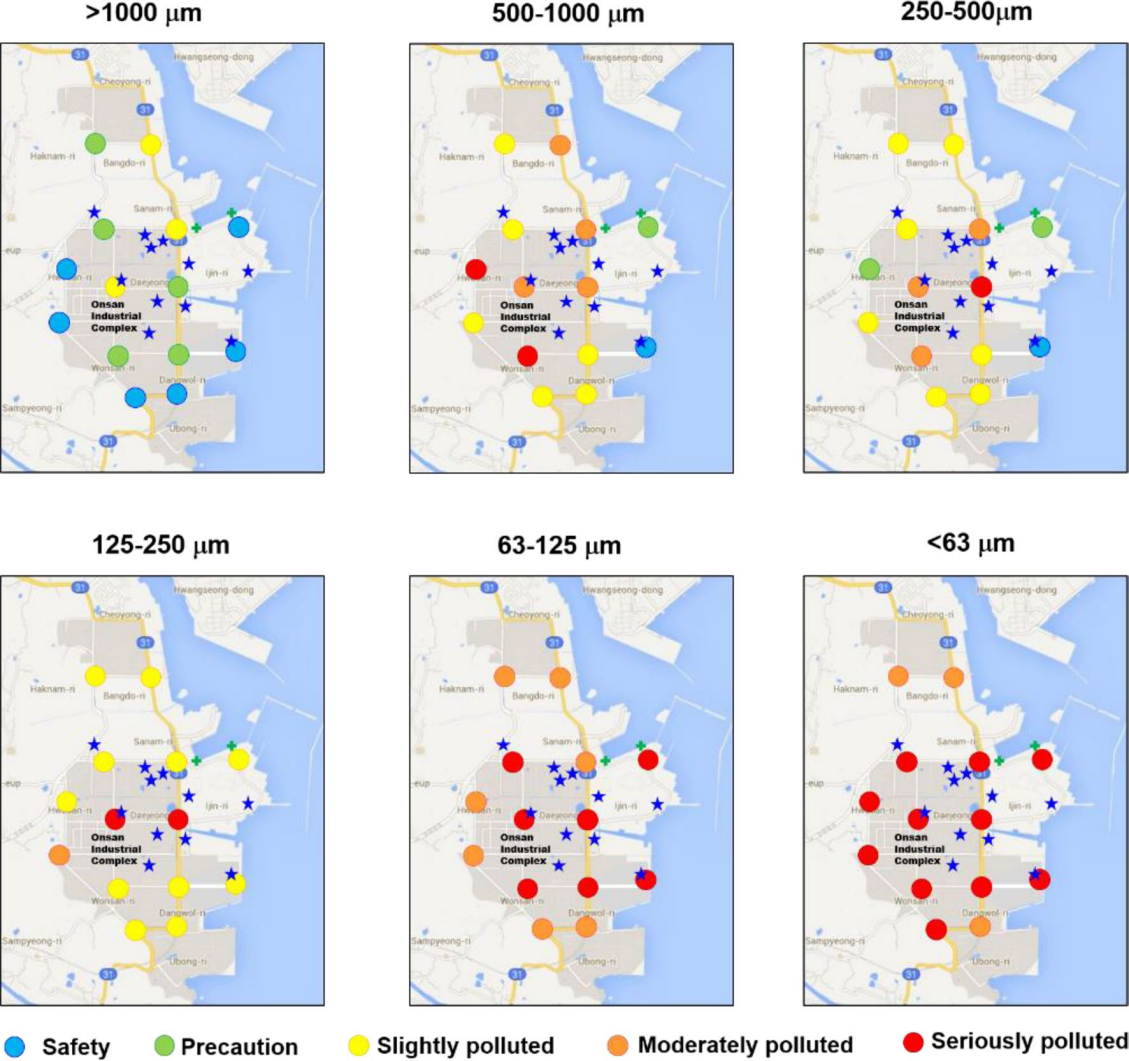
Spatial distribution of nemerow index (P_N_) values in the different sizes of road deposited sediments. Blue star symbol means a smelting facility. Figure created using Microsoft Office PowerPoint 2016 based on Google Maps (available at https://www.google.com/maps).

Figure 4 shows the spatial distribution of P_N_ values in the different sizes of RDS. The spatial distribution of P_N_ values for < 125 μm was high around the smelting facilities, but relatively low at the sampling sites away from the smelters. The high pollution degree of RDS (< 125 μm) indicates that the fine particles of RDS are attached to the tires according to vehicle transport and spreads through the entire road surface. Additionally, the chimney of smelter and vehicle emissions are other potential sources of PTEs in RDS. Bennett and Knapp^63^ reported that the median particle size emitted from Cu, Zn, and Pb smelter ranged from 0.1 to 2.2 μm. The particle size emitted by engine combustion of a vehicle is very small in the size range of 20–150 nm^64^. Given the RDS amounts and spatial distribution of PTEs deposited in the road surface and, the major cause of PTEs contamination in RDS of this study is probably due to spillage and diffusion of raw ore minerals during transportation rather than particulate emissions from smelters.

### Ecological risk assessment in industrial RDS

The results of single factor ecological risk degree (${\text{E}}_{{\text{r}}}^{{\text{i}}}$) are presented in Table 4. The highest mean ${\text{E}}_{{\text{r}}}^{{\text{i}}}$ value was observed for Cd (75,044) in < 63 μm of RDS and the lowest ${\text{E}}_{{\text{r}}}^{{\text{i}}}$ value was observed for Cr (2.6) in > 1000 μm of RDS. Similar to the PTEs concentrations, the single ecological risk was higher as the particle size of RDS decreased. The mean of single factor ecological risk degree (${\text{E}}_{{\text{r}}}^{{\text{i}}}$) values of Cr and Ni in all particle sizes was less than 40, which indicated that Cr and Ni concentrations of RDS correspond to the low ecological risk level. The mean values of ${\text{E}}_{{\text{r}}}^{{\text{i}}}$ of Cd were the highest among those of all PTEs for all sampling sites and ranged from 2095 (> 1000 μm) to 75,044 (< 63 μm), indicating extremely potential risk levels (${\text{E}}_{{\text{r}}}^{{\text{i}}}$ > 320). Hg has the second highest ${\text{E}}_{{\text{r}}}^{{\text{i}}}$ values and exceed 320 in all particle sizes of RDS, showing extremely potential risk. For Cu and Pb, the mean of ${\text{E}}_{{\text{r}}}^{{\text{i}}}$ values were also obtained extremely potential risk except for the large RDS size > 1000 μm. Generally, the ${\text{E}}_{{\text{r}}}^{{\text{i}}}$ values were ranked in the following order: Cd > Hg > Pb > Cu > As > Zn > Ni > Cr. The mean of PER values, the comprehensive ecological risk of eight PTEs, ranged from 4434 (> 1000 μm) to 96,435 (< 63 μm) and the fine particle was 21.7 times higher that large particle. The PER values exceeded 600, indicating very high ecological risk for all studied sites and particle size of RDS except for > 1000 μm at S11 site (Fig. 3).

**Table 4:**
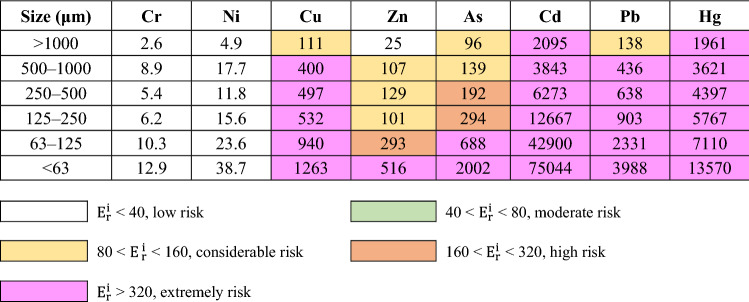
Mean values of single factor ecological risk degree (${\text{E}}_{{\text{r}}}^{{\text{i}}}$) of PTEs in the different size of road deposited sediments.

### PTEs loads in RDS on the road surface around the active smelting industry

**Figure 5:**
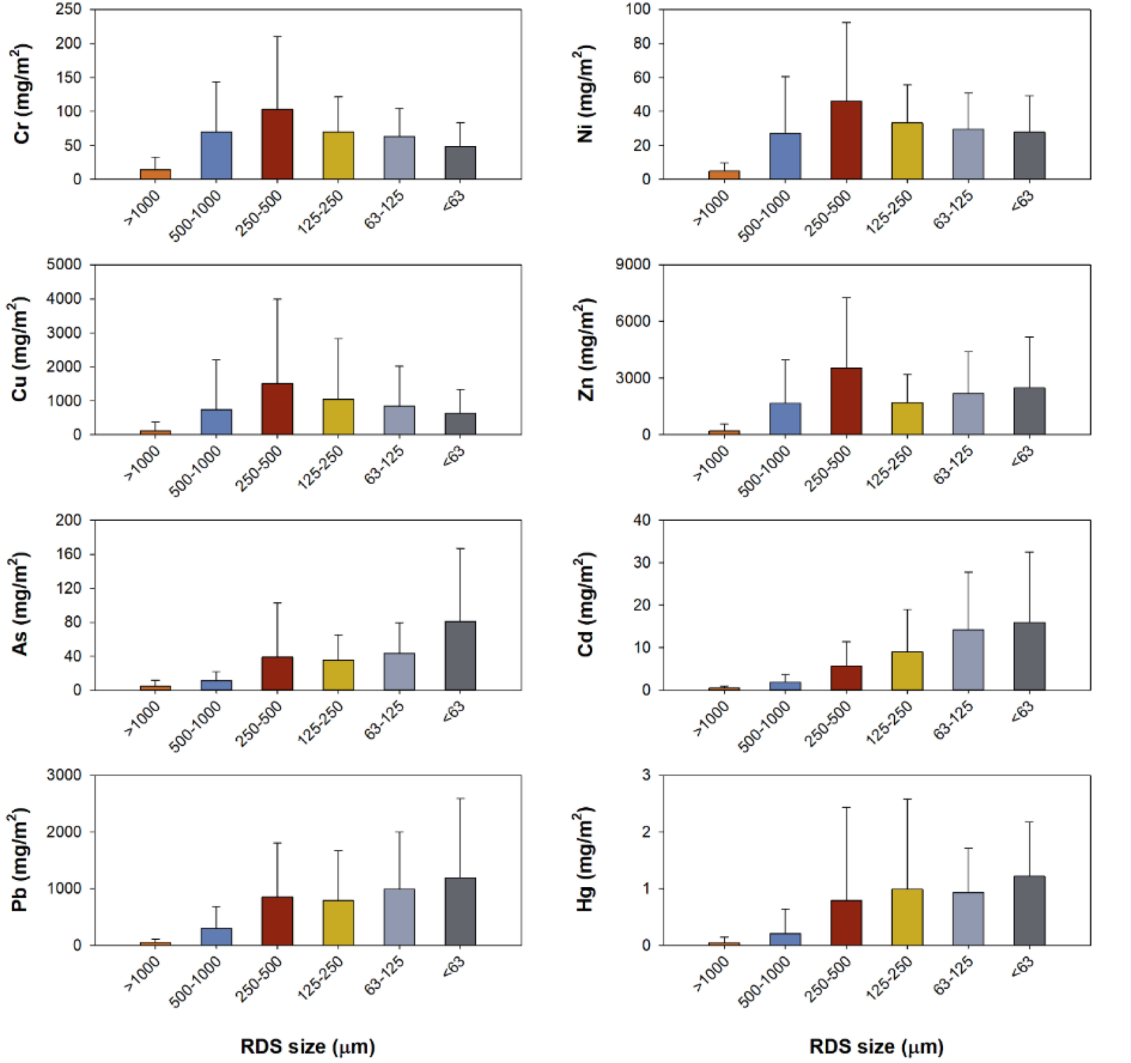
Comparison of mean PTEs loads (mg/m^2^) with standard deviation in the different sizes of road deposited sediments of this study.

**Figure 6:**
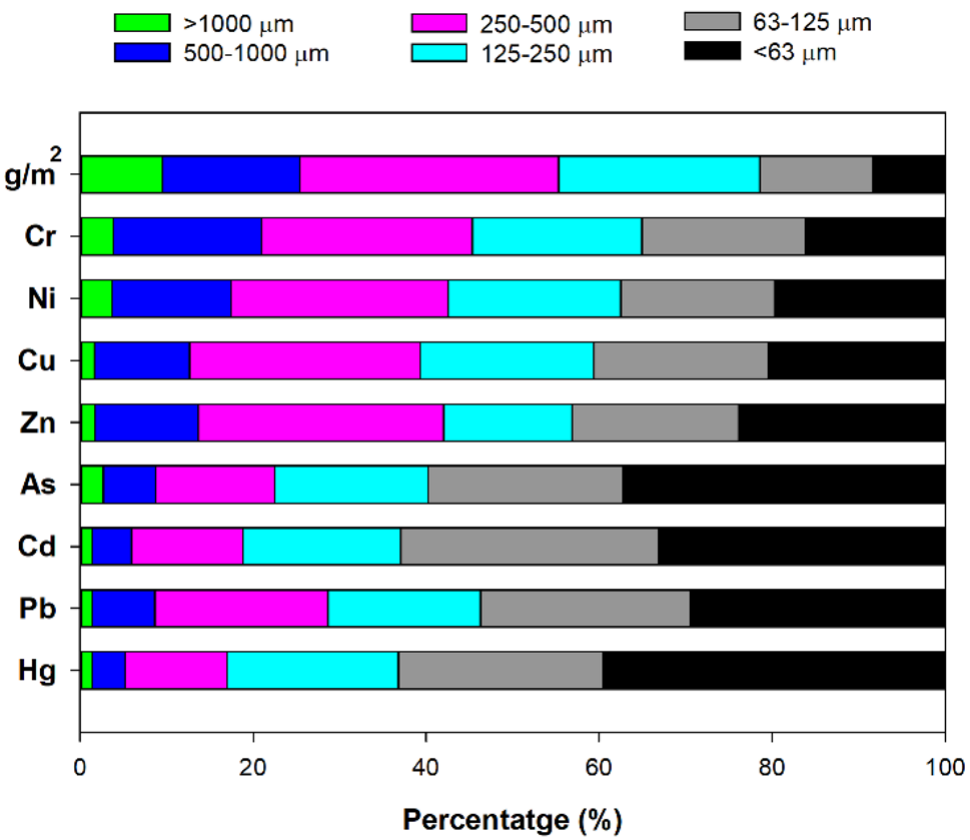
PTEs loading percentages of different sizes of road deposited sediments in this study.

The mean of RDS amount in road surface were 104 for > 1000 μm, 200 for 500–1000 μm, 373 for 250–500 μm, 272 for 125–250 μm, 138 for 63–125 μm, 85 g/m^2^ for < 63 μm, respectively (Table 1). Spatial distribution of amounts in different particle sizes of RDS is shown in Fig. S4. The amount of RDS with particle size of 250–500 μm was the most abundant in this study. We also calculated the PTEs loads and the contribution of each particle size fraction using GSF_loading_ (Fig. 5). A significant amount of PTEs (21,872 mg/m^2^) has accumulated on the road surface in industrial area. The each PTEs load in industrial RDS was much higher than in urban RDS^17^. The order of the sum PTEs loading value in RDS for all measured PTEs was less than 63 μm (26.3%) > 250–500 μm (23.6%) > 63–125 μm (22.5%) > 125–250 μm (16.7%) > 500–1000 μm (9.6%) > above 1000 μm (1.3%). Among the eight PTEs, Zn had the highest GSF_loading_ value per unit area (11,802 mg/m^2^) of road surface, in the order of Cu (4984) > Pb (4177) > Cr (370) > As (215) > Ni (169) > Cd (47) > Hg (4). Given the GSF_loading_ and PTEs concentrations, particles of 250–500 mm showed the highest contribution for Cr, Ni, Cu and Zn, but the mean values of GSF_loading_ were dominant in the < 63 μm fraction for As, Cd, Pb, and Hg (Fig. 6).

The mean of PTEs loading in RDS has accumulated about 48.8% in the < 125 μm fraction, which is readily washed from stormwater runoff and is difficult to remove by road cleaning. Jeong et al.^6^ evaluated the particle size distribution in total suspended solids (TSS) of industrial runoff and found that < 125 μm particle size in TSS ranged from 53.9% to 98.7%. The particle size of < 125 μm RDS accounted for 35.1%, 37.1%, 40.6%, 43.1%, 59.8%, 62.9%, 53.7%, and 63.2 of Cr, Ni, Cu, Zn, As, Cd, Pb, and Hg in total RDS, respectively. Our previous study proposed that RDS make a significant contribution of PTEs pollution to total suspended particles in stormwater runoff at industrial areas^6^.

Road surface is a pollution hotspot where enormous PTEs accumulate in RDS and transport to surrounding environments via stormwater runoff and wind. The curb is the most RDS-accumulated area on a road surface^6,65^. Therefore, road and street sweeping technique is recognized as being an efficient and important tool to reduce stormwater and atmosphere pollution derived from the RDS^66–68^. Tobin and Brinkmann^66^ reported that the rotary brush sweeper is more efficient than a vacuum sweeper for large sediments in the road, but the vacuum sweeper can be effective in removing fine particles. Kim et al.^67^ estimated the removal efficiency of RDS by sweeping with vacuum-assisted rotary brush sweeper in Korea. They found that the mean of reduction in the load of RDS and heavy metals of highway by sweeping was 61.1% and 48%, respectively. The removal of particles (> 63 μm) is greatly improving the highway runoff quality by vacuum-related rotary brush sweeper of RDS, indicating that the sweeping is more efficient for large particles. Given the total length of entire road, the amount of RDS and PTEs concentrations on the road surface, huge amounts of PTEs can be accumulated in the RDS of the industrial area. RDS had the highest concentrations of PTEs in fine particles that are difficult to remove by road sweeping. In Korea, RDS is periodically removed by various types of road cleaning vehicles in urban cities, but road cleaning is not performed in industrial areas. Our results show that road cleaning in industrial areas can remove enormous PTEs that affect the environments and human health. RDS management strategies for fine particles are required to reduce the PTEs pollution and the ecological environmental risk.

## Conclusions

RDS is highly polluted by various pollutants, especially PTEs, and has received much attention as one of the important pollution sources in the terrestrial, coastal, and atmospheric environments as well as human health problems. We studied the concentrations and loadings of PTEs in different particle sizes of RDS around the active smelting industry to figure out their pollution source and to assess the pollution and potential ecological risk levels. PTE concentration in RDS increased with decreasing in particle size and the fine size (< 63 μm) of RDS was heavily polluted with PTEs. Mean metal concentrations (mg/kg) in the fine size (< 63 μm) were on the order of Zn (34,592) > Pb (13,561) > Cu (7071) > As (961) > Cr (596) > Ni (364) > Cd (225) > Hg (17). These concentrations of PTEs in this study were the highest values compared to the soils around the smelter and the RDS in urban cities in the world. The PTEs in RDS could be derived from both particulate emissions from chimney and truck spills during the transportation of raw ore for smelting activity. The spatial distribution of PTEs for < 125 μm was high around the smelting facilities, but relatively low at the sampling sites away from the smelters. Our results indicate that the PTEs in RDS might be affected by spillage and diffusion of raw ore minerals during transportation rather than particulate emission from the smelters. Road surface around the smelter has a significant amount of RDS accumulated with a mean of 21,678 mg/m^2^ compared to urban areas. Cr, Ni, Cu, Zn, As, Cd, Pb, and Hg were accumulated per unit area in amounts of 370, 169, 4984, 11,802, 215, 47, 4177, and 4 mg/m^2^ in the road surface of the study area. The relative contributions of Zn, As, Cd, Pb and Hg in the fraction (< 125 μm) that could transport to the surrounding environments via runoff and resuspension accounted for 39.6% (Zn), 57.9% (As), 63.8% (Cd), 52.3% (Pb) and 51.3% (Hg) of the total RDS. Given the amount of PTEs deposited on the road surface, it is necessary to apply an RDS removal management plan to reduce the PTEs pollution.

## Supplementary Information

Supplementary Information.

## Data Availability

All Data for this study are available from the corresponding author on request.

## References

[1] Adachi K, Tainosho Y (2004). Characterization of heavy metal particle embedded in tire dust. Environ. Int..

[2] Shafer MM, Toner BM, Overdier JT, Schauer JJ, Fakra SC, Hu S, Herner JD, Ayala A (2012). Chemical speciation of vanadium in particulate matter emitted from diesel vehicles and urban atmospheric aerosols. Environ. Sci. Technol..

[3] Adamiec E, Jarosz- Krzemińska E, Wieszała R (2016). Heavy metals from non-exhaust vehicle emissions in urban and motorway road dusts. Environ. Monit. Assess..

[4] Loganathan P, Vigneswaran S, Kandasamy J (2013). Road-deposited sediment pollutants: A critical review of their characteristics, source apportionment, and management. Crit. Rev. Environ. Sci. Technol..

[5] Baensch-Baltruschat B, Kocher B, Stock F, Reifferscheid G (2020). Tyre and road wear particles (TRWP)—A review of generation, properties, emissions, human health risk, ecotoxicity, and fate in the environment. Sci. Total Environ..

[6] Jeong H, Choi JY, Lee J, Lim J, Ra K (2020). Heavy metal pollution by road-deposited sediments and its contribution to total suspended solids in rainfall runoff from intensive industrial areas. Environ. Pollut..

[7] Zhao H, Yin C, Chen M, Wang W (2009). Risk assessment of heavy metals in street dust particles to a stream network. Soil Sediment Contam..

[8] Zhao H, Chen X, Hao S, Jiang Y, Zhao J, Zou C, Xie W (2016). Is the wash-off process of road-deposited sediment source limited or transport limited?. Sci. Total. Environ..

[9] Yuen JQ, Olin PH, Lim HS, Benner SG, Sutherland RA, Ziegler AD (2012). Accumulation of potentially toxic elements in road deposited sediments. J. Environ. Monit..

[10] Jeong H, Choi JY, Lim J, Shim WJ, Kim YO, Ra K (2020). Characterization of the contribution of road deposited sediments to the contamination of the close marine environment with trace metals: Case of the port city of Busan (South Korea). Mar. Pollut. Bull..

[11] Zhu W, Bian B, Li L (2008). Heavy metal contamination of road-deposited sediments in a medium size of China. Environ. Monit. Assess..

[12] Zhao H, Wang X, Li X (2017). Quantifying grain-size variability of metal pollutants in road-deposited sediments using the coefficient of variation. Int. J. Environ. Res. Public Health.

[13] Lanzerstorfer C (2018). Heavy metals in the finest size fractions of road-deposited sediments. Environ. Pollut..

[14] Davis B, Birch G (2010). Comparison of heavy metal loads in stormwater runoff from major and minor urban roads using pollutants yield rating curves. Environ. Pollut..

[15] Du Y, Gao B, Zhou H, Ju X, Hao H, Yin S (2013). Health risk assessment of heavy metals in road dusts in urban parks of Beijing, China. Procedia Environ. Sci..

[16] Roy S, Gupta SK, Prakash J, Habib G, Baudh K, Nasr M (2019). Ecological and human health risk assessment of heavy metal contamination in road dust in the National Capital Territory (NCT) of Delhi, India. Environ. Sci. Pollut. Res..

[17] Zhao H, Li X, Wang X, Tian D (2010). Grain size distribution of road-deposited sediments and its contribution of heavy metal pollution in urban runoff in Beijing, China. J. Hazard. Mater..

[18] Hilliges R, Endres M, Tiffert A, Brenner E, Marks T (2016). Characterization of road runoff with regards to seasonal variations, particle size distribution and the correlation of fine particles and pollutants. Water Sci. Technol..

[19] Charlesworth S, De Miguel E, Ordóñez A (2011). A review of the distribution of particle trace elements in urban terrestrial environments and its application to considerations of risk. Environ. Geochem. Health.

[20] Li HH, Chen LJ, Yu L, Gou ZB, Shan CQ, Lin JQ, Gu YG, Yang ZB, Yang YX, Shao JR, Zhu XM, Cheng Z (2017). Pollution characteristics and risk assessment of human exposure to oral bioaccessibility of heavy metals via urban street dusts from different functional areas in Chengdu, China. Sci. Total Environ..

[21] Wu L, Luo XS, Li H, Cang L, Yang J, Yang J, Zhao Z, Tang M (2019). Seasonal levels, sources, and health risks of heavy metals in atmospheric PM_2.5_ from four functional areas of Nanjing city, eastern China. Atmosphere.

[22] Jeong H, Choi JY, Ra K (2020). Study on heavy metal pollution sources to Shihwa lake: characteristics of heavy metal in size-fractionated road dust from urban area and the impacts to marine environments. J. Korean Soc. Mar. Environ. Energy.

[23] Choi JY, Jeong H, Choi KY, Hong GH, Yang DB, Kim K, Ra K (2020). Source identification and implications of heavy metals in urban roads for the coastal pollution in a beach town, Busan, Korea. Mar. Pollut. Bull..

[24] Das R, Khezri B, Srivastava B, Datta S, Sikdar PK, Webster RD, Wang X (2015). Trace element composition of PM_2.5_ and PM_10_ from Kolkata-a heavily polluted Indian Metropolis. Atmos. Pollut. Res..

[25] Hwang HM, Fiala MJ, Park D, Wade TL (2016). Review of pollutants in urban road dust and stormwater runoff: Part 1. Heavy metals released from vehicles. Int. J. Urban Sci..

[26] Aguilera A, Armendariz C, Quintana P, García-Oliva F, Bautista F (2019). Influence of land use and road type on the elemental composition of urban dust in a Mexican Metropolitan Area. Pol. J. Environ. Stud..

[27] Wentworth CK (1992). A scale of grade and class terms for clastic sediments. J. Geol..

[28] Muller G (1969). Index of geoaccumulation in sediments of the Rhine River. GeoJournal.

[29] Rudnick, R.I. & Gao, S. Composition of the continental crust. In: Rudnick, R.L., editor. *The Crust*, Elsevier, pp. 1–64 (2003).

[30] Liu J, Wu J, Feng W, Li X (2020). Ecological risk assessment of heavy metals in water bodies around typical copper mines in China. Int. J. Environ. Res. Public Health.

[31] Nemerow, N.L. *Stream, Lake, Estuary, and Ocean Pollution *(1991).

[32] Yang Z, Lu W, Long Y, Bao X, Yang Q (2011). Assessment of heavy metals contamination in urban topsoil from Changchun City, China. J. Geochem. Explor..

[33] Nezhad MTK, Tabatabaii SM, Gholami A (2015). Geochemical assessment of steel smelter-impacted urban soils, Ahvaz, Iran. J. Geochem. Explor..

[34] Huang L, Rad S, Xu L, Gui L, Song X, Li Y, Wu Z, Chen Z (2020). Heavy metals distribution, sources, and ecological risk assessment in Huixian wetland, South China. Water.

[35] Men C, Liu R, Xu L, Wang Q, Guo L, Miao Y, Shen Z (2020). Source-specific ecological risk analysis and critical source identification of heavy metals in road dust in Beijing, China. J. Hazard. Mater..

[36] Hong-gui D, Teng-feng G, Ming-hui L, Xu D (2012). Comprehensive assessment model on heavy metal pollution in soil. Int. J. Electrochem. Sci..

[37] Ministry of Government Legislation. Korea soil quality standard of heavy metals in soil environment conservation act (Law No. 16613) (2019).

[38] Lazo P (2009). Determination of Cr (VI) in environmental samples evaluating Cr (VI) impact in a contaminated area. J. Int. Environ. Appl. Sci..

[39] Jie-liang C, Zhou S, You-Wei Z (2007). Assessment and mapping of environmental quality in agricultural soils of Zhejiang Province, China. J. Environ. Sci..

[40] Hakanson L (1980). An ecological risk index for aquatic pollution control. A sedimentological approach. Water Res..

[41] Lu X, Wu X, Wang Y, Chen H, Gao P, Fu Y (2014). Risk assessment of toxic metals in street dust from a medium-sized industrial city of China. Ecotoxicol. Environ. Saf..

[42] Zhao W, Ding L, Gu X, Luo J, Liu Y, Guo L, Shi Y, Huang T, Cheng S (2015). Levels and ecological risk assessment of metals in soils from a typical e-waste recycling region in southeast China. Ecotoxicol..

[43] Feng Y, Bao Q, Yunpeng C, Lizi Z, Xiao X (2019). Stochastic potential ecological risk model for heavy metal contamination in sediment. Ecol. Indic..

[44] Sutherland RA (2003). Lead in grain size fractions of road-deposited sediment. Environ. Pollut..

[45] Garmash GA (1985). Distribution of heavy metals in soils near metallurgical plants. Soviet Soil Sci..

[46] Wang K, Tian H, Hua S, Zhu C, Gao J, Xue Y, Hao J, Wang Y, Zhou J (2016). A comprehensive emission inventory of multiple air pollutants from iron and steel industry in China: Temporal trends and spatial variation characteristics. Sci. Total Environ..

[47] Jo., M., Lee, M. & Kim, K.R. Investigation of soil Cr and Ni contamination in different land uses and tracing the source of contamination. *Korean J. Soil Sci. Fert.* **53**, 510–518 (2020) **(in Korean)**.

[48] Zgłobicki W, Telecka M, Skupiński S (2019). Assessment of short-term changes in street dust pollution with heavy metals in Lublin (E Poland)—Levels, sources and risks. Environ. Sci. Pollut. Res..

[49] Jeong H, Choi JY, Ra K (2020). Assessment of metal pollution of road-deposited sediments and marine sediments around Gwangyang Bay, Korea. J. Korean Soc. Oceanogr..

[50] Duong TTT, Lee BK, Dong TTT, Jeong U, Kim A, Lee HK (2011). Heavy metal contamination of road dust at the downtown area in the Metropolitan city of Ulsan, Korea. J. Environ. Manag..

[51] Zafra CA, Temprano J, Tejero I (2011). Distribution of the concentration of heavy metals associated with the sediment particles accumulated on road surface. Environ. Technol..

[52] Kachenko AG, Singh B (2006). Heavy metals contamination in vegetables grown in urban and metal smelter contaminated sites in Australia. Water Air Soil Pollut..

[53] Denaix L, Semlali RM, Douay F (2001). Dissolved and colloidal transport of Cd, Pb, and Zn in a silt loam affected by atmospheric industrial deposition. Environ. Pollut..

[54] Kierczak J, Potysz A, Pietranik A, Tyszka T, Modelska M, Néel C, Ettler V, Mihaljevič M (2013). Environmental impact of the historical Cu smelting in the Rudawy Janowickie Mountains (south-western Poland). J. Geochem. Explor..

[55] Verner JF, Ramsey MH, Helios-Rybicka E, Jêdrzejczyk B (1996). Heavy metal contamination of soils around a Pb-Zn smelter in Bukowno, Poland. Appl. Geochem..

[56] Nahmani J, Hodson ME, Black S (2007). Effects of metals on life cycle parameters of the earthworm *Eisenia fetida* exposed to field-contaminated, metal-polluted soils. Environ. Pollut..

[57] Bacon JR, Dinev NS (2005). Isotopic characterisation of lead in contaminated soils from the vicinity of a non-ferrous metal smelter near Plovdiv, Bulgaria. Environ. Pollut..

[58] Li Z, Feng X, Li G, Bi X, Sun G, Zhu J, Qin H, Wang J (2011). Mercury and other metal and metalloid soil contamination near a Pb/Zn smelter in east Hunan province, China. Appl. Geochem..

[59] Cui YJ, Zhu YG, Zhai RH, Chen DY, Huang YZ, Qiu Y, Liang JZ (2004). Transfer of metals from soil to vegetables in an area near a smelter in Nanning, China. Environ. Pollut..

[60] Šajn R, Aliu M, Stafilov T, Alijagić J (2013). Heavy metal contamination of topsoil around a lead and zinc smelter in Kosovska Mitrovica/Mitrovicë, Kosovo/Kosovë. J. Geochem. Explor..

[61] Stafilov T, Šajn R, Pančevski Z, Boev B, Frontasyeva MV, Strelkova LP (2010). Heavy metal contamination of topsoils around a lead and zinc smelter in the Republic of Macedonia. J. Hazard. Mater..

[62] Mihaljevič M, Ettler V, Vaněk A, Penížek V, Svoboda M, Kříbek B, Sracek O, Mapani BS, Kamona AF (2015). Trace elements and the lead isotopic record in marula (*Sclerocarya birrea*) tree rings and soils near the Tsumeb Smelter, Namibia. Water Air Soil Pollut..

[63] Bennett RL, Kanpp KT (1989). Characterization of particulate emissions from non-ferrous smelters. JAPCA.

[64] Agarwal AK, Gupta T, Lukose J, Singh AP (2015). Particulate characterization and size distribution in the exhaust of a gasoline homogeneous charge compression ignition engine. Aerosol Air Qual. Res..

[65] Gustafsson M, Blomqvist G, Järlskog I, Lundberg J, Janhäll S, Elmgren M, Johansson C, Norman M, Silvergren S (2019). Road dust load dynamics and influencing factors for six winter seasons in Stockholm, Sweden. Atmos. Environ. X.

[66] Tobin GA, Brinkmann R (2002). The effectiveness of street sweepers in removing pollutants from road surfaces in Florida. J. Environ. Sci. Health.

[67] Kim DG, Jeong K, Ko SO (2014). Removal of road deposited sediments by sweeping and its contribution to highway runoff quality in Korea. Environ. Technol..

[68] Polukarova M, Markiewicz A, Björklund K, Strömvall AM, Galfi H, Sköld YA, Gustafsson M, Järlskog I, Aronsson M (2020). Organic pollutants, nano- and microparticles in street sweeping road dust and washwater. Environ. Int..

