# Supplementary material for: Potentially toxic elements pollution in road deposited sediments around the active smelting industry of Korea
Source: Sci Rep. 2021 Mar 31;11:7238. doi: 10.1038/s41598-021-86698-x (PMC8012626; doi:10.1038/s41598-021-86698-x)

**[Supplementary materials]**

**Potentially toxic elements pollution in road deposited sediments around the active smelting industry of Korea**

Hyeryeong Jeong^1,2^, Jin Young Choi^1^, and Kongtae Ra^1,2,⸸^

^1^Marine Environmental Research Center, Korea Institute of Ocean Science & Technology (KIOST), Busan 49111, Korea
^2^Department of Ocean Science (Oceanography), KIOST School, University of Science & Technology (UST), Daejeon 34113, Korea
^⸸^corresponding.author: ktra@kiost.ac.kr

**Table S1.** Minimum, maximum, and mean values of Al, Fe, Ti and Li concentration in the different sizes of road-deposited sediments of this study.

| **Size** |  | **Al** | **Fe** | **Ti** | **Li** |
| --- | --- | --- | --- | --- | --- |
| (μm) |  | % | % | % | mg/kg |
| >1000 | Min | 5.90 | 2.54 | 0.24 | 30.4 |
|  | Max | 9.18 | 8.98 | 0.42 | 45.5 |
|  | **Mean** | **6.82** | **5.19** | **0.33** | **3.70** |
|  | SD | 0.83 | 2.29 | 0.06 | 5.3 |
| 500~  1000 | Min | 4.28 | 2.63 | 0.16 | 20.2 |
|  | Max | 7.06 | 17.29 | 0.34 | 38.5 |
|  | **Mean** | **5.40** | **10.10** | **0.25** | **30.9** |
|  | SD | 0.79 | 4.65 | 0.05 | 5.7 |
| 250~  500 | Min | 3.69 | 2.93 | 0.14 | 17.6 |
|  | Max | 5.44 | 14.93 | 0.42 | 31.3 |
|  | **Mean** | **4.66** | **9.62** | **0.23** | **24.4** |
|  | SD | 0.54 | 4.13 | 0.08 | 3.8 |
| 125~  250 | Min | 3.02 | 2.95 | 0.27 | 13.8 |
|  | Max | 5.71 | 11.74 | 2.53 | 27.1 |
|  | **Mean** | **4.60** | **7.07** | **0.55** | **20.0** |
|  | SD | 0.70 | 2.46 | 0.59 | 4.0 |
| 63~  125 | Min | 3.43 | 4.73 | 0.29 | 13.4 |
|  | Max | 6.80 | 11.21 | 1.73 | 27.7 |
|  | **Mean** | **5.56** | **8.14** | **0.62** | **22.2** |
|  | SD | 0.94 | 2.10 | 0.43 | 3.9 |
| <63 | Min | 3.37 | 6.28 | 0.20 | 13.3 |
|  | Max | 7.56 | 12.71 | 1.19 | 31.5 |
|  | **Mean** | **5.82** | **8.68** | **0.51** | **24.5** |
|  | SD | 1.24 | 1.74 | 0.23 | 5.4 |

**Table S2.** Principal component analysis (PCA) for potentially toxic elements investigated in this study. Factor loadings in bold type are >0.7

|  | Component | |
| --- | --- | --- |
|  | PC1 | PC2 |
| Cr | 0.070 | **0.947** |
| Ni | 0.087 | **0.957** |
| Cu | **0.823** | 0.029 |
| Zn | **0.922** | -0.016 |
| As | **0.892** | 0.170 |
| Cd | **0.937** | 0.006 |
| Pb | **0.713** | 0.333 |
| Hg | 0.350 | 0.038 |
| Initial eigenvalues | 4.003 | 1.797 |
| % of variance | 50.034 | 22.464 |
| Cumulative % | 50.034 | 72.498 |


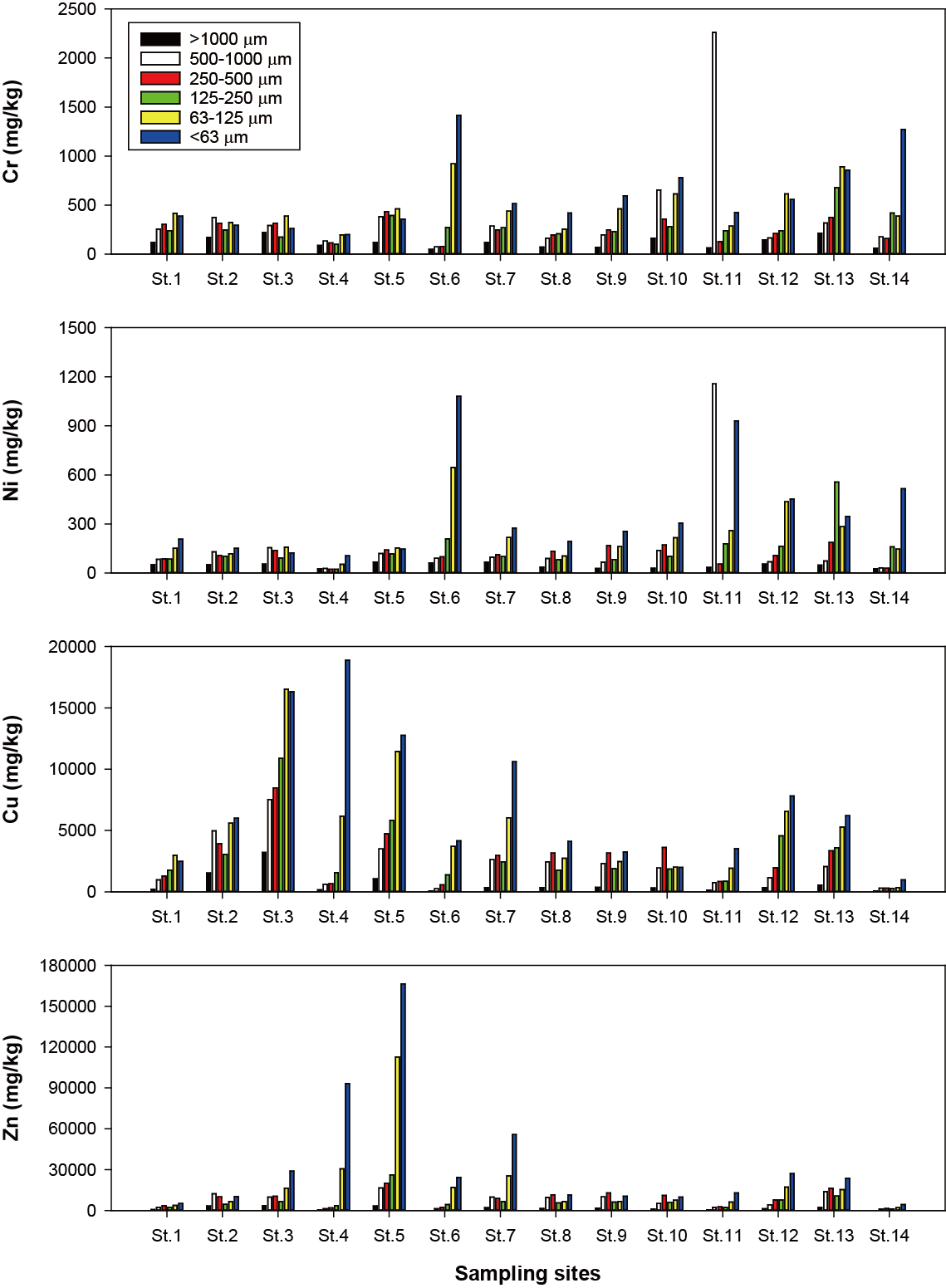
 **Fig. S1.** Spatial distributions of Cr, Ni, Cu, and Zn (mg/kg) in different particle sizes of road deposited sediments.


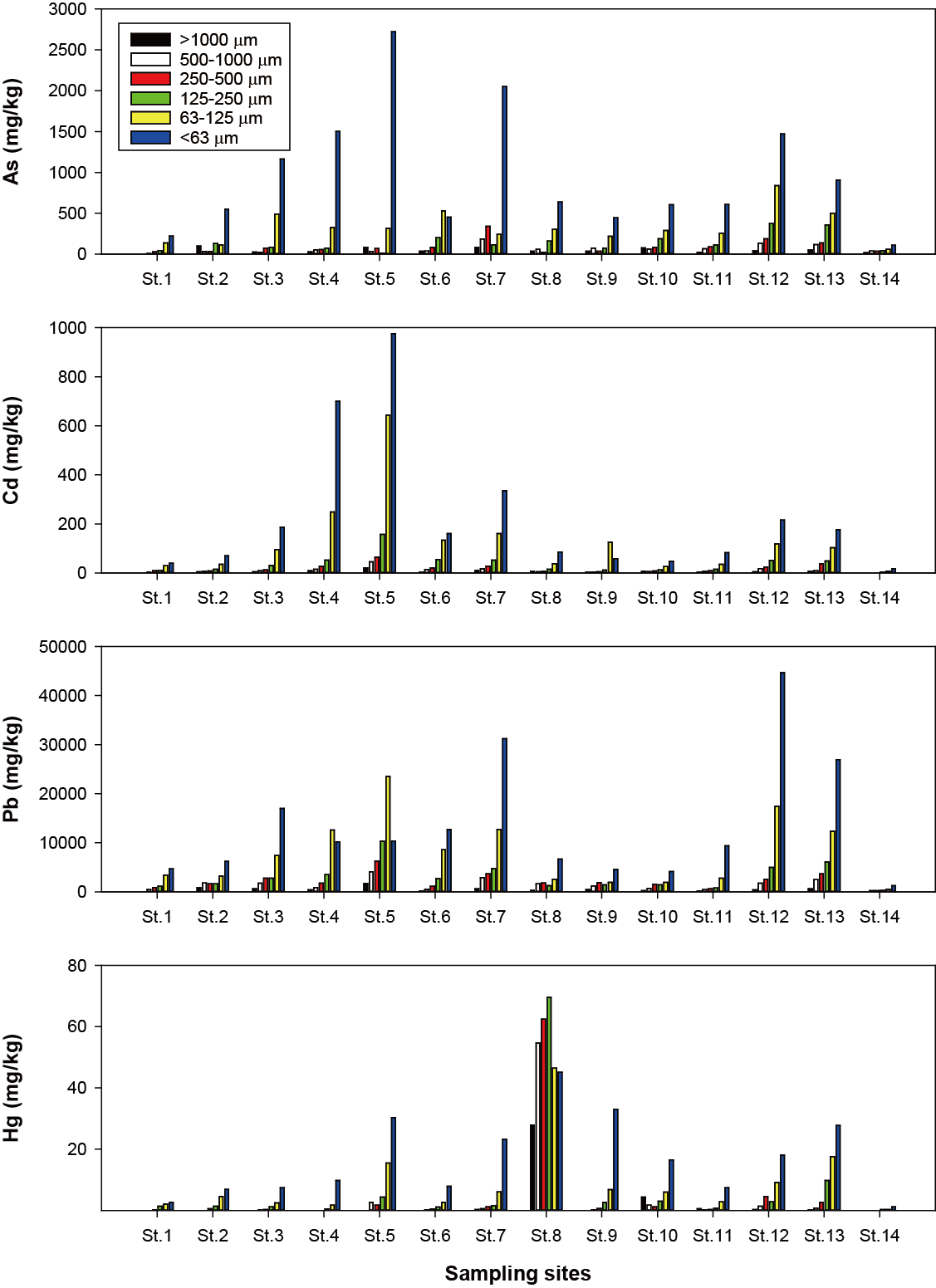


**Fig. S2.** Spatial distributions of As, Cd, Pb and Hg (mg/kg) in different particle sizes of road deposited sediments.


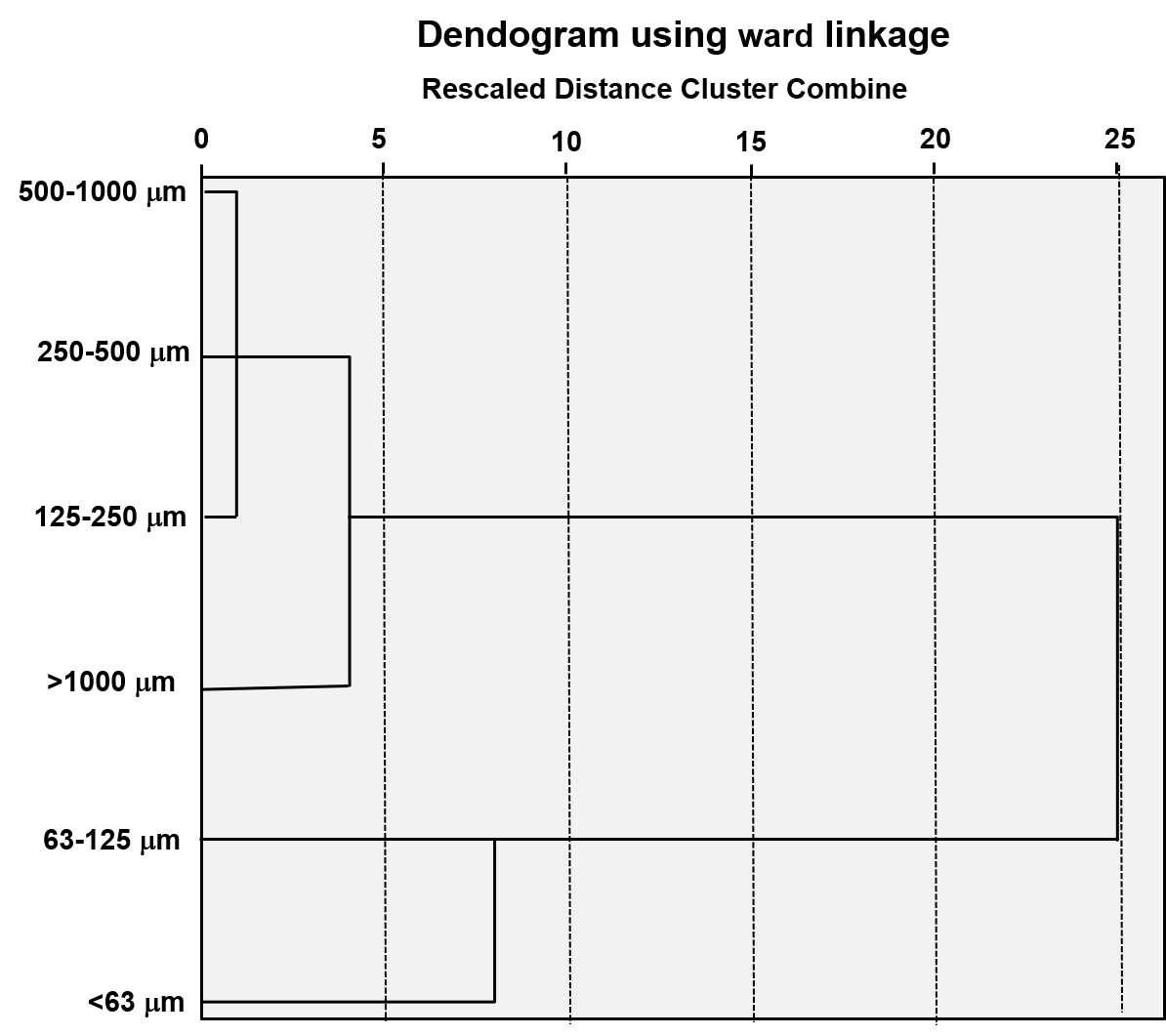


**Fig. S3.** Dendrogram showing clustering in different particle sizes of road deposited sediments.

**Fig. S4** Spatial distribution of amounts (g/m^2^) in different particle sizes of road deposited sediments in this study.


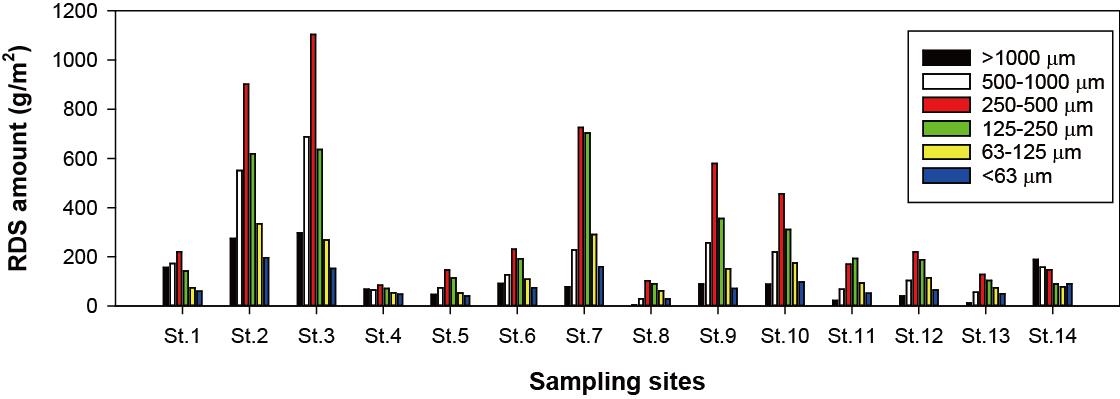

Supplement: Supplementary file 1 — Supplementary Information. [file 41598_2021_86698_MOESM1_ESM.docx]
